# The triterpenoid CDDO-imidazolide ameliorates mouse liver ischemia-reperfusion injury through activating the Nrf2/HO-1 pathway enhanced autophagy

**DOI:** 10.1038/cddis.2017.386

**Published:** 2017-08-10

**Authors:** Dongwei Xu, Lili Chen, Xiaosong Chen, Yankai Wen, Chang Yu, Jufang Yao, Hailong Wu, Xin Wang, Qiang Xia, Xiaoni Kong

**Affiliations:** 1 Department of Liver Surgery, Renji Hospital, School of Medicine, Shanghai Jiao Tong University, Shanghai, China; 2 School of Biomedical Engineering and Med-X Research Institute, Shanghai Jiao Tong University, Shanghai, China; 3 Animal Laboratory, Renji Hospital, School of Medicine, Shanghai Jiao Tong University, Shanghai, China; 4 State Key Laboratory of Cell Biology, CAS Center for Excellence in Molecular Cell Science, Innovation Center for Cell Signaling Network, Institute of Biochemistry and Cell Biology, Shanghai Institutes for Biological Sciences, Chinese Academy of Sciences, Shanghai, China

## Abstract

Nuclear factor erythroid 2-related factor 2 (Nrf2)-mediated induction of antioxidants has been implicated to have protective roles in ischemia-reperfusion (I/R) injury in many animal models. However, the *in vivo* effects of CDDO-imidazole (CDDO-Im) (1-[2-cyano-3-,12-dioxooleana-1,9(11)-dien-28-oyl] imidazole), a Nrf2 activator, in hepatic I/R injury is lacking and its exact molecular mechanisms are still not very clear. The goals of this study were to determine whether CDDO-Im can prevent liver injury induced by I/R in the mouse, and to elucidate the molecular target of drug action. Mice were randomly equally divided into two groups and administered intraperitoneally with either DMSO control or CDDO-Im (2 mg/kg) 3 h before subjected to 90-min hepatic 70% ischemia followed by reperfusion. Subsequently, the Liver and blood samples of these mice were collected to evaluate liver injury. CDDO-Im pretreatment markedly improve hepatic I/R injury by attenuating hepatic necrosis and apoptosis, reducing reactive oxygen species (ROS) levels and inflammatory responses, and ameliorating mitochondrial dysfunction. Mechanistically, by using Nrf2 Knockout mice and hemeoxygenase 1 (HO-1) inhibitor, we found that these CDDO-Im protection effects are attributed to enhanced autophagy, which is mediated by activating Nrf2/HO-1 pathway. By accelerating autophagy and clearance of damaged mitochondria, CDDO-Im reduced the mtDNA release and ROS overproduction, and in turn decreased damage-associated molecular patterns induced inflammatory responses and the following secondary liver injury. These results indicate that by enhancing autophagy, CDDO-Im-mediated activation of Nrf2/HO-1 signaling could be a novel therapeutic strategy to minimize the adverse effects of hepatic I/R injury.

Ischemia-reperfusion (I/R) injury widely exists in clinical procedures such as liver resection, organ transplantation, trauma and shock. Hepatic I/R injury remains an important issue affecting the long-term graft survival of patients with liver transplantation, and accounts for ~10% of early postoperative liver failure and at least 10% of the acute or chronic rejection.^1, 2^ However, there are still no effective drugs to protect the liver from I/R injury so far. During the ischemic process, the absence of oxygen supplement and the consumption of glycogen lead to ATP metabolic disorder, mitochondrial dysfunction and initial of cell death. During the reperfusion process, activation of the Kupffer cells and neutrophils result in the production of lots of inflammatory cytokines and reactive oxygen species (ROS), leading to enhanced liver injury.^3^

Nuclear factor erythroid 2-related factor 2 (Nrf2) is a master transcription factor in regulating antioxidant production to maintain cellular redox homeostasis.^4, 5, 6^ Under normal circumstances, Nrf2 is located in the cytoplasm and degraded by its repressor Kelch-like erythroid-associated protein 1 (Keap1) through the ubiquitination and proteasome pathway. Although in pathological conditions, the interaction between Keap1 and Nrf2 is disrupted by numerous stimuli and activated Nrf2 is translocated into the nucleus to promote the transcription of many relevant cytoprotective and antioxidant genes such as NAD(P)H quinone dehydrogenase 1 and hemeoxygenase 1 (HO-1) via binding to the antioxidant response elements (AREs) in promoter regions of those genes.^4^ Previous studies have demonstrated that Nrf2 deficiency donor liver exacerbated IR injury in transplant recipients, whereas activation of Nrf2 has hepatoprotective effects.^7, 8, 9^ Furthermore, pharmacologic activation of Nrf2 has demonstrated to increase hepatic GSH concentrations, and therefore increase bile flow, which may be able to counteract cholestatic conditions and modify oxidative stress and inflammation in these diseases progression.^9, 10, 11^ CDDO-imidazolide (CDDO-Im), one of the synthetic oleanane triterpenoids, is a potent activator of the Nrf2 pathway and proved to be a protective agent in a number of disease models, including acute kidney, lung or neurons injury, acetaminophen hepatotoxicity, emphysema and sepsis.^12, 13, 14, 15, 16, 17^ Here, we hypothesized that CDDO-Im may protect hepatic I/R injury by activating Nrf2 pathway.

Autophagy is a cytoprotective process involving in degradation of long-lived cytoplasmic proteins, surplus or dysfunctional organelles through a lysosome-dependent machinery to maintain cellular homeostasis.^18^ Numerous studies have shown that autophagy has crucial roles in liver pathology such as metabolic diseases, infectious diseases and hepatocellular carcinoma.^19^ So far the role of autophagy in the pathogenesis of I/R injury is controversial.^20, 21^ Our previous study indicates that induction of autophagy protects liver from I/R injury.^22^ Here, we further demonstrate that CDDO-Im protects hepatic I/R injury by activating the Nrf2-HO-1-autophagy axis.

In this study, we investigated the role of CDDO-Im in hepatic I/R injury and demonstrated that CDDO-Im could ameliorate liver I/R injury by activating the Nrf2/HO-1 pathway and induction of autophagy, which is responsible for clearance of damaged mitochondria and reduced production of ROS and proinflammatory cytokines. Given that mitochondrial dysfunction is a major early detrimental event contributing to induction of high ROS and inflammatory response in I/R injury, CDDO-Im preconditioning may be a potent therapeutic approach to target this early stage of I/R injury process.

## Results

### CDDO-Im protects liver from I/R injury

**Figure 1:**
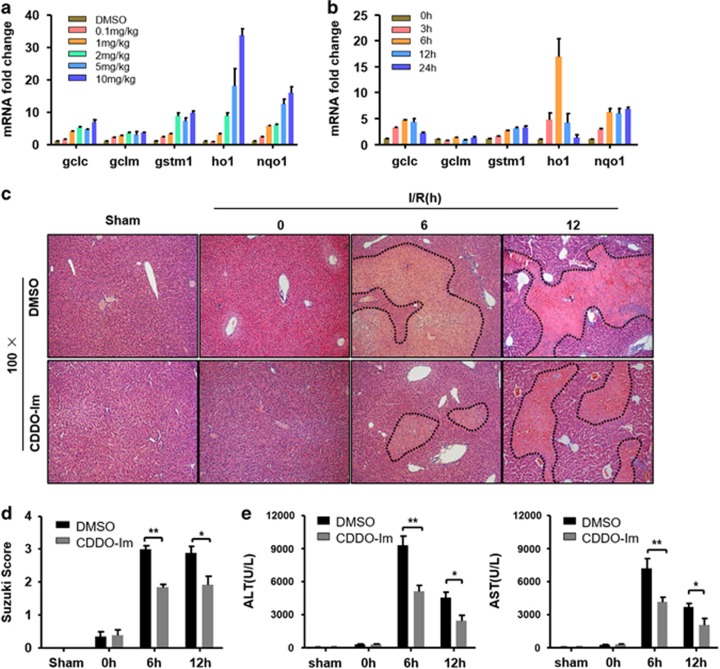
CDDO-Im pretreatment attenuates I/R injury. (**a**,**b**) Time-course (0–24 h) and dose-response (0–10 mg/kg) of CDDO-Im pretreatment and Nrf2 target genes mRNA levels (*n*=3 per group). (**c**) Representative hematoxylin and eosin (HE)-stained sections (original magnification, × 100) and relevant average Suzuki score (**d**) at 0, 6, 12 h post reperfusion or sham controls. (mean±S.E.M., *n*=4–6 per group). (**e**) Serum ALT and AST levels at 0, 6, 12 h post reperfusion; mean±S.E.M., *n*=4–6 per group. **P<0.01, *P<0.05

**Figure 2:**
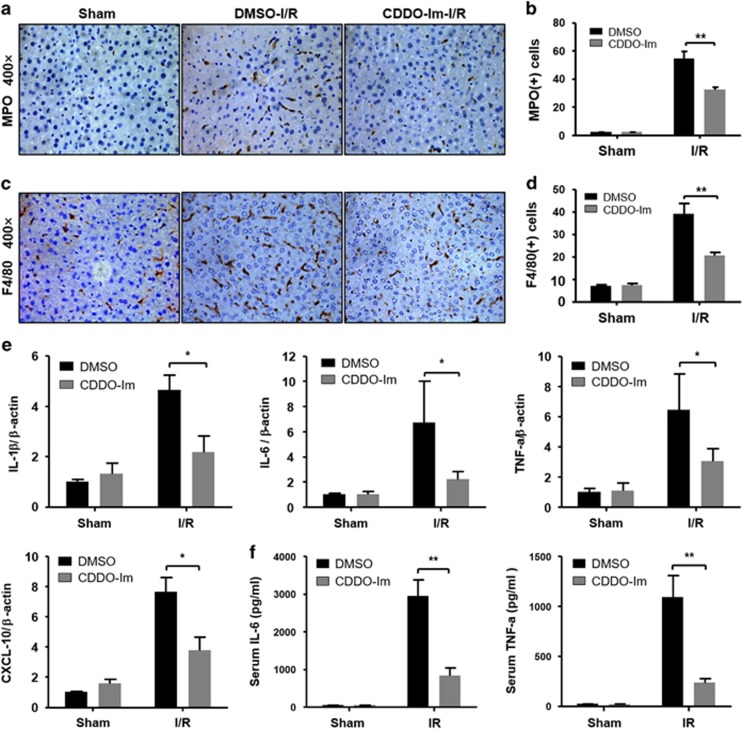
CDDO-Im depresses neutrophil infiltration and reduces cytokine release. (**a**) Representative sections from MPO staining and F4/80 staining (**c**) at 6 h after reperfusion (original magnification, × 400), the numbers of MPO (**b**) and F4/80 (**d**) positive cells that infiltrated the livers were determined. (**e**) mRNA levels of cytokines and chemokines were determined by quantitative real-time PCR, and (**f**) Serum cytokine levels measured by ELISA. All data shown as the mean±S.E.M., **P<0.01, *P<0.05

CDDO-Im is a well-known activator of Nrf2.^13, 17^ To assess whether CDDO-Im treatment has a protective role on liver I/R injury, we started to determine the proper treatment dose and time of CDDO-Im in wild-type (WT) mice by measuring the transcriptional induction of Nrf2 target genes such as *Gclc, Gclm, Gstm1, Ho1* and *Nqo1* in liver tissues. As shown in Figures 1a and b, 2 mg/kg of CDDO-Im treatment for 3 h could significantly increase the hepatic expression of those genes. In our liver I/R model, mice pretreated with CDDO-Im or vehicle were subjected to 90-mi hepatic 70% ischemia, followed by reperfusion and the liver I/R injury was assessed by biochemical measurements and histopathology analyses. As shown in Figure 1c, compared with mice pretreated with vehicle whose hepatic necrotic areas were markedly increased 6 h and 12 h post reperfusion, CDDO-Im pretreated mice showed great resistance to I/R-induced liver injury. In line with the decreased liver injury, the liver sections of CDDO-Im pretreated mice were graded with significantly lower Suzuki scores than those in the vehicle group (Figure 1d). Consistent with these histological alterations, CDDO-Im pretreatment also markedly decreased serum aspartate aminotransferase (AST) and alanine transaminase (ALT) levels (Figure 1e). These results clearly demonstrated that CDDO-Im pretreatment attenuates the detrimental effects of hepatic I/R injury.

### CDDO-Im alleviates inflammatory responses during hepatic I/R injury

Given that the activation of the inflammatory response plays an important role in exacerbating hepatic I/R injury, we started to assess the accumulation of hepatic inflammatory cells by staining myeloperoxidase (MPO) (a neutrophil marker) and F4/80 (a macrophage marker) after I/R injury. As shown in Figures 2a and d, CDDO-Im treatment resulted in significantly decreased hepatic infiltration of both neutrophils and macrophages after reperfusion compared with the vehicle control group. In response to the reduced hepatic infiltration of innate immune cells, significantly less induction of proinflammatory cytokines such as IL-1*β*, IL-6, TNF-*α* and CXCL-10 was detected in the CDDO-Im group compared with the vehicle group 6 h after I/R treatment (Figures 2e and f). These findings clearly demonstrated that CDDO-Im pretreatment alleviates the inflammatory responses following hepatic I/R treatment.

### CDDO-Im reduces hepatocyte apoptosis in hepatic I/R injury

**Figure 3:**
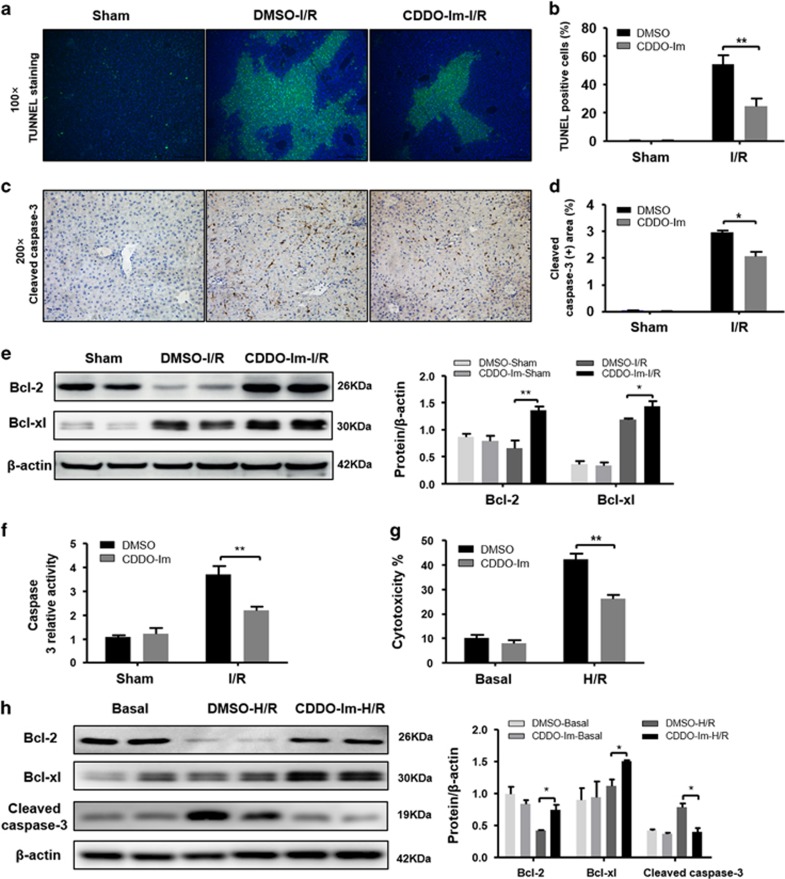
CDDO-Im pretreatment alleviate apoptosis in hepatic I/R injury both *in vitro* and vivo. (**a**,**b**) Representative sections of TUNEL staining at 6 h after reperfusion. TUNEL-positive hepatocytes were expressed as a percentage of the total hepatocytes. (**c**,**d**) Representative sections from cleaved caspase-3 staining (original magnification, × 200), the cleaved caspase-3-positive area that infiltrated the livers were determined. (**e**) Western blot analysis of Bcl2 and Bcl-xl in the livers of DMSO and CDDO-Im pretreatment mice at 6 h after I/R or a sham operation. *β*-actin served as a loading control. (**f**) Caspase-3 activity was evaluated from a total lysate of I/R lobes or sham mice from CDDO-Im or DMSO pretreated group. (*n*=3–4 per group, mean±S.E.M., **P<0.01, *P<0.05). (**g**) Cell death of isolated primary hepatocytes after H/R injury were measured by LDH assays. Average cytotoxicity (% cell death) in different groups were plotted. Three to five replicates per experiment group. (**h**) Bcl2, Bcl-xl and cleaved caspase-3 protein expression were analyzed by western blotting in isolated hepatocytes from WT with or without CDDO-Im pretreatment subjected to H/R for 4 h. (*n*=3–4 per group, mean±S.E.M., **P<0.01, *P<0.05)

As I/R injury is associated with liver apoptosis, which is mainly mediated by TNF-α,^23^ we then assessed the hepatic apoptosis levels in both CDDO-Im-treated and untreated groups by terminal deoxynucleotidyl transferase dUTP nick end labeling (TUNEL) assays. Although I/R induced marked DNA damages in both groups, CDDO-Im treated mice displayed significantly reduced TUNEL signals in the liver sections compared with control mice (Figures 3a and b). In line with the TUNEL assay results, immunohistochemistry (IHC) staining of cleaved caspase-3 also showed decreased signals in the CDDO-Im treated group compared with the untreated one (Figures 3c and d). This reduced apoptosis in CDDO-Im treated mice is at least partially due to increased expression of anti-apoptotic proteins, such as Bcl2 and Bcl-xl because western blot showed an increase of those two genes in the CDDO-Im treated group compared with the control group (Figure 3e). In addition, the caspase-3 activities were markedly decreased in the CDDO-Im group compared with the control group (Figure 3f). Given that previous studies have reported the positive regulation of Nrf2 on the expression of Bcl2 and Bcl-xl,^24^ it is not surprising to detect elevated Bcl2 and Bcl-xl levels in the CDDO-Im treated group. To further confirm the protective effect of CDDO-Im on hepatocyte apoptosis during I/R injury, we isolated primary hepatocytes from WT mice and pretreated them with or without CDDO-Im *in vitro* followed with hypoxia/reoxygenation (H/R) to mimic *in vivo* I/R injuries. H/R-induced cell damage was determined by Lactate dehydrogenase (LDH) cytotoxicity assays. In line with our *in vivo* findings, compared with the control group, CDDO-Im pretreatment significantly preserved hepatocytes in H/R treatment as demonstrated by reduced LDH release (Figure 3g). This protection might be due to diminished apoptosis levels, because reduced cleaved caspase-3 and increased Bcl2 and Bcl-xl levels were detected in the hepatocytes treated with CDDO-Im after H/R (Figure 3h). Therefore, these results clearly demonstrated that CDDO-Im protects hepatocytes against I/R induced apoptosis.

### CDDO-Im enhances liver autophagy during hepatic I/R injury

**Figure 4:**
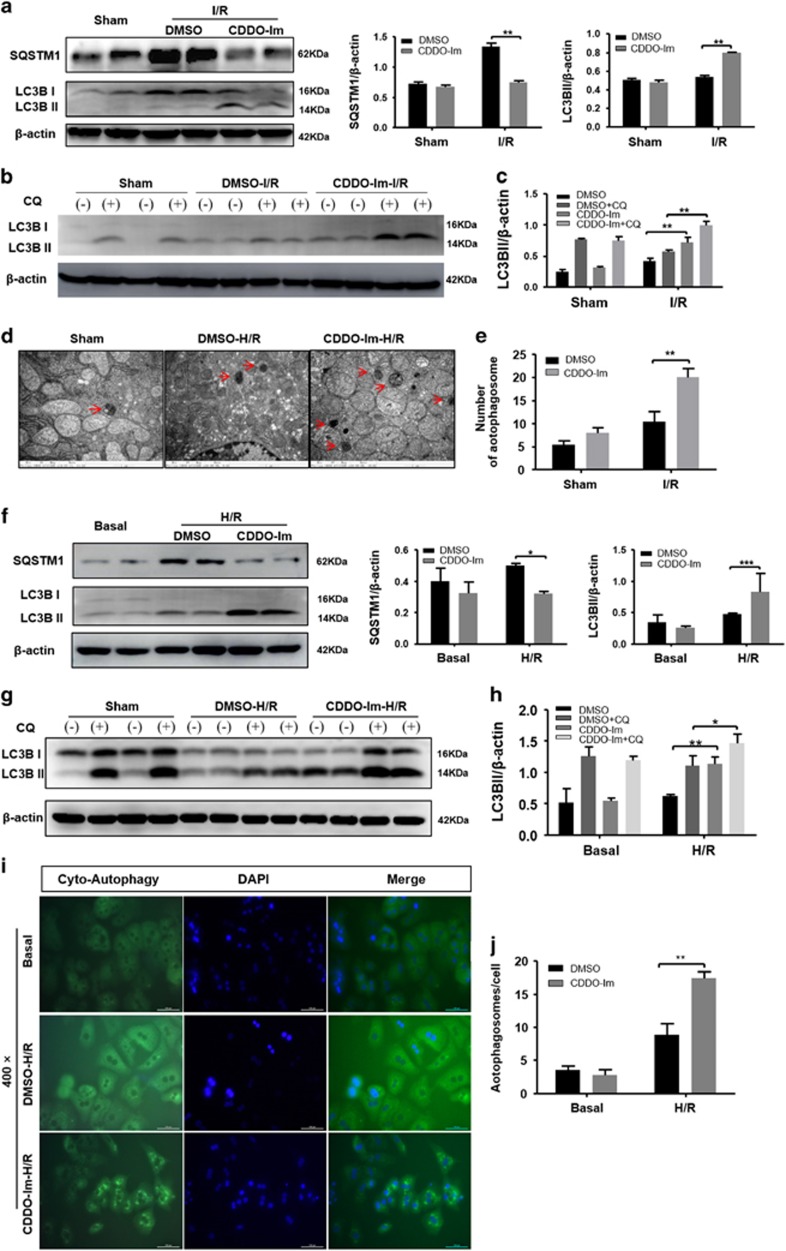
CDDO-Im pretreatment enhances liver autophagy induction in hepatic I/R injury. (**a**) Western blot analysis of LC3B and SQSTM1 protein expression in the CDDO-Im and DMSO pretreatment group at 6 h after reperfusion. *β*-actin served as a loading control. Coresponding densitometric analysis of LC3B-II and SQSTM1 expression. (**b**,**c**) Western blot analysis of LC3B protein expression in the presence and absence of chloroquine (60 mg/kg) and densitometric analysis of LC3B-II expression. *β*-actin was used as the loading control. (**d**,**e**) Representative transmission electron micrographs showing autophagosomes in the ischemic lobes at 6 h of reperfusion. Autophagosomes are indicated by arrows. Scale bars 1 *μ*m. The numbers of autophagosomes were determined. The data are shown as the mean±S.E.M. *n*=4-6 per group. (**f**) Western blot analysis of LC3B and SQSTM1 protein expression in the CDDO-Im and DMSO pretreatment primary hepatocytes isolated from WT, which subjected to H/R injury. *β*-actin served as a loading control. Coresponding densitometric analysis of LC3B-II and SQTM1 expression. (**g**,**h**) Western blot analysis of LC3B protein expression in the presence and absence of chloroquine (50 mM) and densitometric analysis of LC3B-II expression. *β*-actin was used as the loading control. (**i,j**) Representative fluorescence micrographs display autophagosomes in hepatocytes with CDDO-Im and DMSO pretreatment (original magnification, × 400). The numbers of autophagosomes were determined. The data are shown as the mean±S.E.M. *n*=4-6 per group

Autophagy has emerged recently as an important safeguard against I/R injury by clean-up damaged mitochondria and in turn inhibiting excessive ROS production.^25^ We then sought to investigate whether CDDO-Im affects autophagy during hepatic I/R injury. As shown in Figure 4a, levels of LC3b-II, a standard indicator of autophagy activity, were greater in the CDDO-Im pretreated mice than that in the control mice 6 h after I/R injury. Moreover, compared with the controls, CDDO-Im pretreatment resulted in a marked decrease in the levels of SQSTM1/p62, a classic macroautophagy substrate (Figure 4a). Autophagy is a highly dynamic and multi-step process. Therefore, LC3b-II levels at certain time point are insufficient to represent autophagy flux. We then employed Chloroquine (CQ) to block autophagy flux in mice with or without CDDO-Im pretreatment. Although CQ treatment significantly increased the LC3B-II levels in both groups, CDDO-Im pretreated mice showed greater LC3B-II elevation as compared with controls (Figures 4b and c). Correspondingly, analysis of autophagy using transmission electron microscope (TEM) showed increased numbers of autophagosomes in the liver of CDDO-Im pretreatment mice compared with the control mice 6 h after I/R injury (Figures 4d and e). Meanwhile, we similarly investigated the effect of CDDO-Im on autophagy induction in primary hepatocytes *in vitro* after H/R injury. In line with our *in vivo* findings, compared with DMSO control, CDDO-Im treatment significantly elevated autophagy activity during H/R injury as evidenced by substantially increased LC3B-II levels and decreased SQSTM1/p62 levels (Figure 4f). CQ-mediated autophagy flux blocking assays also showed enhanced autophagy flux in CDDO-Im treated hepatocytes compared with DMSO controls (Figures 4g and h). Meanwhile, accumulation of autophagic vacuoles was detected by CYTO-ID autopahy detection kit. As shown in Figures 4i and j, autophagic vesicles in CDDO-Im pretreatment hepatocytes were more abundant than that in control hepatocytes after H/R injury. These results suggested that CDDO-Im pretreatment enhances autophagy during hepatic I/R injury.

### CDDO-Im protects against mitochondrial dysfunction and excessive ROS induction during liver I/R injury

**Figure 5:**
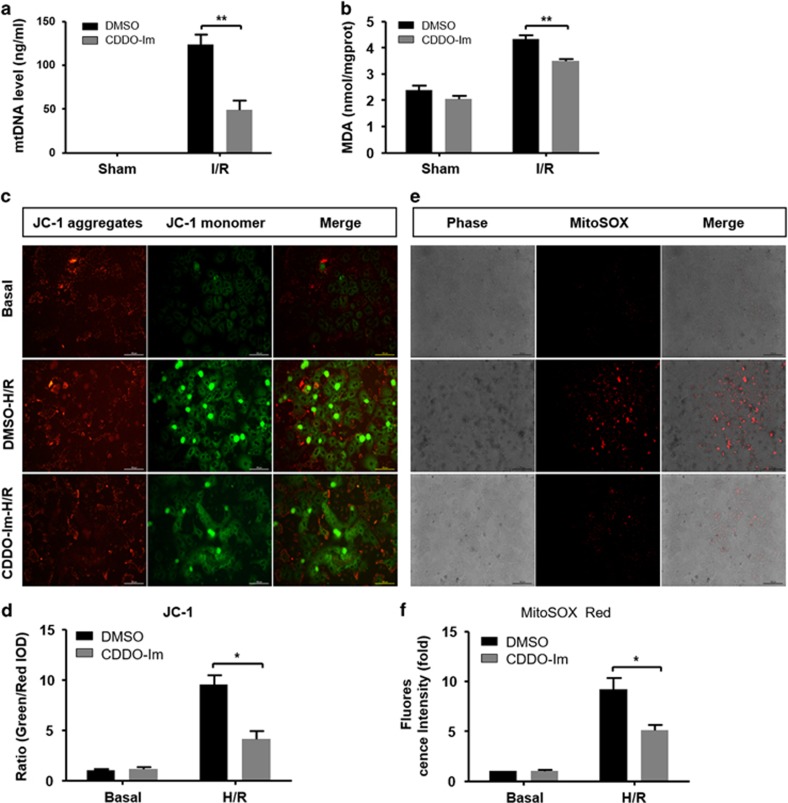
CDDO-Im pretreatment inhibit oxidative stress and protect against mitochondrial dysfunction during hepatic I/R injury. (**a**) Serum levels of mtDNA in mice at 6 h after reperfusion. (**b**) Effects of the CDDO-Im and DMSO on the levels of MDA in mice livers at 6 h after reperfusion. (**c**,**d**) Representative pictures of mitochondrial membrane potential of primary hepatocytes isolated from WT, which subjected to H/R injury in the CDDO-Im and DMSO pretreatment group (original magnification, × 400). The ratio of green to red fluorescence intensity were determined. (**e**,**f**) Representative pictures of mitochondrial ROS accumulation of primary hepatocytes after H/R injury in both groups (original magnification, × 200). The mitochondrial ROS was expressed by relative red area of the total picture. Values expressed as mean±S.E.M. (*n*=4-6). *P<0.05 and **P<0.01

Given the important role of autophagy in removal of damaged and ROS overproduction mitochondria, we accessed the presence of damaged mitochondria in both groups by detecting circulating mitochondrial DNA (mtDNA) in serum. In line with the differential autophagy activities in these two groups, the amount of the circulating mtDNA was markedly lower in CDDO-Im pretreated mice compared with control mice (Figure 5a). In response to the less presence of damaged mitochondria, reduced ROS levels were detected in CDDO-Im pretreated mice as demonstrated by decreased malonaldehyde (MDA) levels (Figure 5b). To further confirm the protective role of CDDO-Im in mitochondrial dysfunction and ROS overproduction, primary hepatocytes from WT mice were treated with H/R injury *in vitro*. Mitochondrial injury and mitochondrial ROS levels were accessed via mitochondrial membrane potential (ΔΨm) (using JC-1 fluorescent dye) and mitochondrial ROS (using mitoSOX Red dye), respectively. As shown in Figures 5c and d, compared with the DMSO control, CDDO-Im treatment displayed protection on ΔΨm as indicated by decreased ratio of green to red fluorescence intensity. In addition, hepatocytes with CDDO-Im treatment showed less mitochondrial ROS production as evidenced by reduced MitoSOX Red fluorescent signals (Figures 5e and f).

### Autophagy accounts for CDDO-Im-mediated protection in liver I/R injury

**Figure 6:**
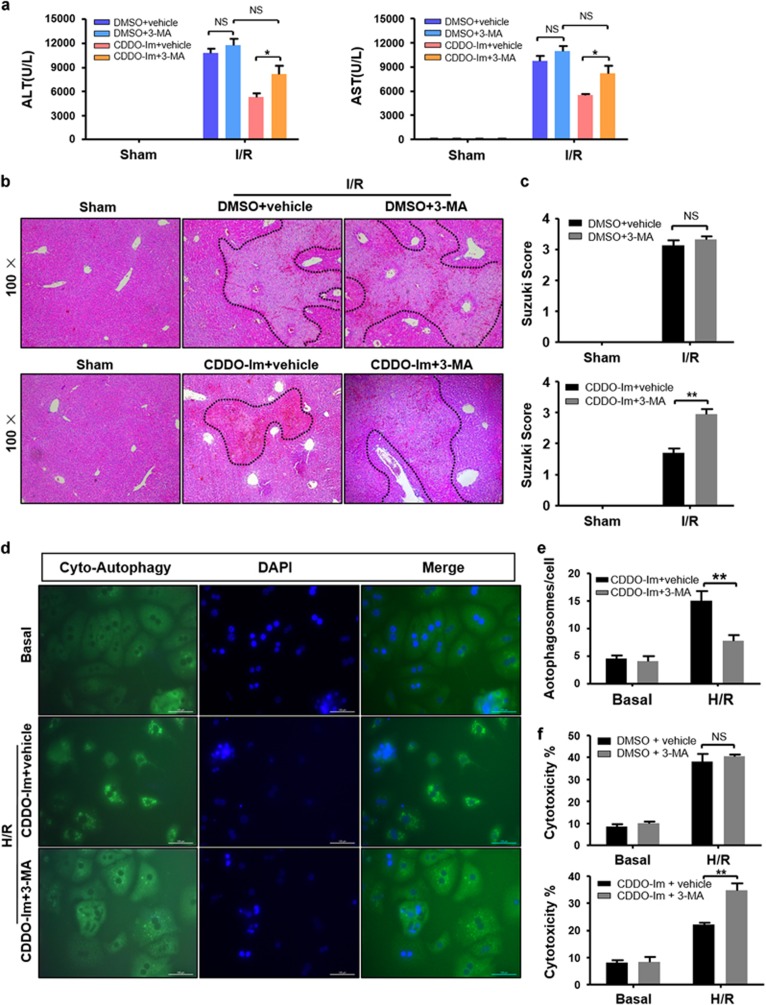
CDDO-Im pretreatment protected against liver I/R injury by means of autophagy induction. (**a–c**) Mice were pretreated with 3-Methyladenine (30 mg/kg, IP) 2 h after CDDO-Im (2 mg/kg, IP) or DMSO and killed at 6 h after reperfusion. (**a**) Serum ALT and AST levels. (**b**) Representative hematoxylin and eosin (HE)-stained sections (original magnification, × 100) and (**c**) relevant average Suzuki score. (**d**, **e**) Representative fluorescence micrographs display autophagosomes in primary hepatocytes with 3-MA in the presence or absence of CDDO-Im (original magnification, × 400). The numbers of autophagosomes were determined. (**f**) Quantification of average cytotoxicity (% cell death) in different groups were plotted. (*n*=3–4 per group, mean±S.E.M., **P<0.01, *P<0.05)

To test whether the cytoprotection of CDDO-Im was mediated by induction of autophagy, we adopted a pharmacological autophagy inhibitor 3-methyladenine (3-MA) to block autophagy in both groups during I/R injury. As shown in Figure 6a, blocking of autophagy remarkably increased the serum ALT/AST levels in the CDDO-Im pretreated group but not in the control group. Consistently, hematoxylin and eosin (HE) staining showed greatly increased necrotic areas in the liver of CDDO-Im pretreated mice owing to autophagy blockage (Figures 6b and c). In addition, 3-MA treatment showed a significant autophagy inhibition in the CDDO-Im group as demonstrated by decreased cellular autophagosomes (Figures 6d and e). Correspondingly, autophagy inhibition resulted in hepatocytes of CDDO-Im group more sensitive to H/R induced cell injury as indicated by increased LDH levels (Figure 6f). These findings clearly indicated that CDDO-Im-mediated hepatic protection against I/R injury mainly depend on autophagy induction.

### CDDO-Im-induced autophagy is dependent on Nrf2/HO-1 Pathway

**Figure 7:**
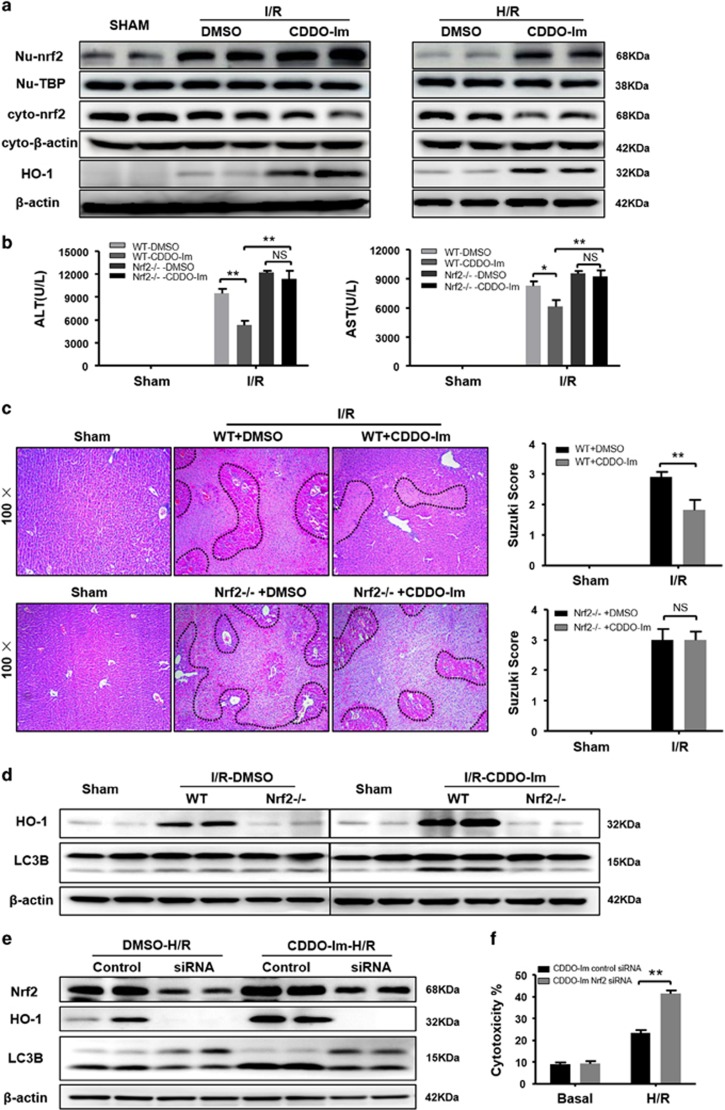
CDDO-Im in induction of autophagy depends on induction of Nrf2/HO-1 pathway. (**a**) Western blot analysis of nuclear and cytoplasm Nrf2 protein expression both in the CDDO-Im and DMSO pretreatment mice livers at 6 h after reperfusion and primary hepatocytes isolated from WT, which subjected to H/R injury. TBP served as a nuclear loading control, whereas *β*-actin served as a cytoplasm loading control. (**b**) WT and Nrf2 KO mice were pretreated with CDDO-Im (2 mg/kg, IP) or DMSO and killed at 6 h after reperfusion. (**b**) Serum ALT and AST levels. (**c**) Representative hematoxylin and eosin(HE) stained sections (original magnification, × 100) and relevant average Suzuki score. (**d**) Western blot analysis of HO-1 and LC3B both in the CDDO-Im and DMSO pretreatment WT and Nrf2 KO mice livers at 6 h after reperfusion. (*n*=3-4 per group, mean±S.E.M., **P<0.01, *P<0.05). (**e**) Immunoblots indicating expression of Nrf2, HO-1 and LC3B protein after treatment with Nrf2 siRNA in the presence and absence of CDDO-Im (200 *μ*M). (**f**) Quantification of average cytotoxicity (% cell death) in different groups were plotted. Three to five replicates per experiment group, mean±S.E.M., **P<0.01, *P<0.05

As CDDO-Im is a well known potent pharmacological Nrf2 activator by promoting nuclear translocation of Nrf2^26^ and HO-1, a target gene of Nrf2, has been reported to reduce liver injury by induction of autophagy via an unknown mechanism,^27, 28^ we further investigated whether CDDO-Im-mediated autophagy is through the Nrf2/HO-1 pathway. I/R injury-mediated Nrf2 activation was confirmed by western blot as indicated by an increase in nuclear Nrf2 levels and a decreased in cytoplasmic Nrf2 levels (Figure 7a). Interestingly, although the nuclear Nrf2 levels seemed comparable in both DMSO control and CDDO-Im groups, CDDO-Im pretreatment substantially enhanced HO-1 expression (Figure 7a). This CDDO-Im pretreatment associated elevation was further confirmed by IHC staining of HO-1 in the liver sections after I/R injury (Supplementary Figure S1A). We next employed Nrf2 knockout (KO) mice to further identify whether CDDO-Im-mediated protection and autophagy are the Nrf2-dependent. Nrf2 depletion abrogated CDDO-Im-mediated protection against liver I/R injury as demonstrated by increased serum ALT/AST levels and necrotic areas (Figures 7b and c). In addition, CDDO-Im-mediated HO-1 induction and autophagy enhancement were dramatically decreased in the Nrf2 KO mice (Figure 7d). To further confirm that COOD-Im-mediated cytoprotection and autophagy are Nrf2-dependent, we performed Nrf2 RNAi in primary hepatocytes followed with H/R injury. As shown in Supplementary Figure S2A, Nrf2 RNAi could efficiently knockdown Nrf2 levels in both the cell nucleus and cytoplasm subsets. In accordance with the *in vivo* results, expression of HO-1 and autophagy activity were remarkably decreased, whereas the H/R induced cytotoxicity was significantly increased in Nrf2 knockdown hepatocytes after CDDO-Im treatment (Figures 7e and f). Therefore, these results clearly demonstrate that CDDO-Im-mediated autophagy and protection against liver I/R injury are dependent on Nrf2.

### CDDO-Im-mediated autophagy depends on induction of HO-1

**Figure 8:**
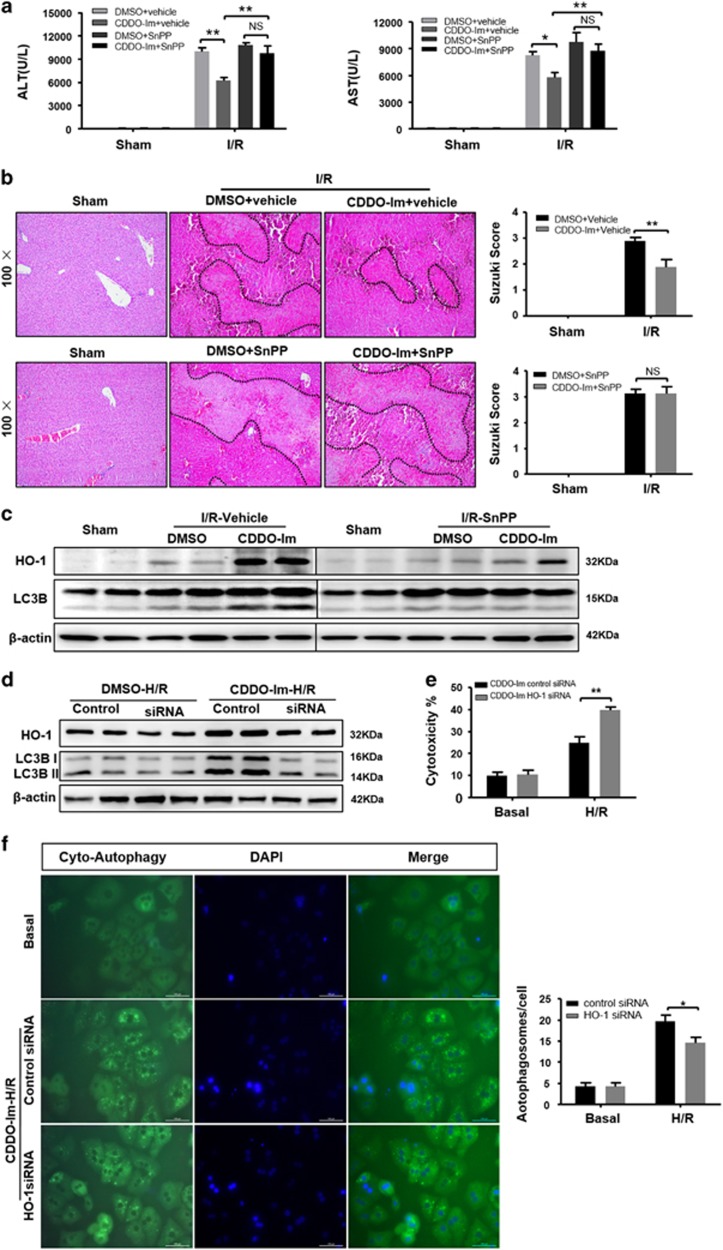
CDDO-Im in induction of autophagy depends on induction of HO-1. (**a**–**c**) WT mice were pretreated with SnPP (50 mg/kg, IP) 1 h before CDDO-Im (2 mg/kg, IP) or DMSO and killed at 6 h after reperfusion. (**a**) Serum ALT and AST levels. (**b**) Representative hematoxylin and eosin (HE) stained sections (original magnification, × 100) and relevant average Suzuki score. (**c**) Western blot analysis of HO-1 and LC3B both in the SnPP and vehicle pretreatment with or without CDDO-Im mice livers at 6 h after reperfusion. (*n*=3-4 per group, mean±S.E.M., **P<0.01, *P<0.05). (**d**) Immunoblots indicating expression of HO-1 and LC3B protein after treatment with HO-1 siRNA in the presence and absence of CDDO-Im (200 *μ*M) and densitometric analysis of LC3B-II and HO-1 expression. (**e**) Quantification of average cytotoxicity (% cell death) in different group were plotted. (**f**) Representative fluorescence micrographs display autophagosomes in primary hepatocytes with CDDO-Im in the presence HO-1 siRNA or control siRNA (original magnification, × 400). The numbers of autophagosomes were determined. (*n*=3-4 per group, mean±S.E.M., **P<0.01, *P<0.05)

To determine whether HO-1 is required for CDDO-Im-mediated autophagy and hepatic protection against I/R injury, we employed a HO-1 inhibitor Tin Protoporphyrin IX dichlorid (SnPP) to treat WT mice followed with I/R injury. Administration of SnPP abolished CDDO-Im-mediated hepatic protection as demonstrate by increased serum ALT/AST levels and necrotic areas (Figures 8a and b), indicated that the protective effects of CDDO-Im were dependent on HO-1. In addition, CDDO-Im-mediated autophagy was also remarkably decreased in SnPP treated mice as indicated by declined LC3B-II levels (Figure 8c). To further confirm that COOD-Im-mediated cytoprotection and autophagy are HO-1-dependent, we performed HO-1 RNAi in primary hepatocytes followed with H/R injury. As shown in Supplementary Figure S2B, HO-1 RNAi efficiently knocked down HO-1 levels. Autophagy activity was significantly decreased in the CDDO-Im group but not the DMSO control as indicated by declined LC3B-II levels (Figure 8d). Meanwhile, the H/R-induced cytotoxicity was significantly increased in HO-1 knockdown hepatocytes after CDDO-Im treatment as evidenced by increased LDH levels (Figure 8e). In addition, impaired autophagy of CDDO-Im group activity was further confirmed by fluorescent staining of autophagosomes (Figure 8f). These findings strongly indicate that CDDO-Im-mediated autophagy induction depends on the activation of Nrf2/HO-1.

## Discussion

In present study, we demonstrated that CDDO-Im can improve I/R induced liver injury. Phenotypically, CDDO-Im attenuates hepatocyte necrosis and apoptosis, reduces inflammatory responses and ameliorates mitochondrial dysfunction during liver I/R injury. These protection phenotypes are attributed to CDDO-Im-mediated autophagy enhancement. Mechanistically, by activating Nrf2 and stimulating its downstream target gene HO-1 expression, CDDO-Im enhances HO-1-mediated autophagy, intensifies the clearance of damaged mitochondria and reduces the mtDNA release and ROS overproduction, and in turn dulled the damage-associated molecular patterns (DAMPs) induced inflammatory responses and the following secondary liver injury.

Although necrosis is conventionally recognized as a main detrimental factor during liver I/R injury, apoptosis has been implicated recently in the pathogenesis of this process. It is well established that mitochondrial dysfunction is a key trigger to initiate cell death by ATP depletion, uncontrolled ROS surge and final mitochondrial breakdown, which finally lead to cell apoptosis and necrosis. In present study, we demonstrate that CDDO-Im could protect both cell apoptosis and necrosis during I/R injury. CDDO-Im-mediated protection on apoptosis maybe partly due to Nrf2 activation and in turn induction of downstream anti-apoptotic genes such as Bcl2 and Bcl-xl. This is consistent with previous studies showing that Nrf2 activation leads to expression of anti-apoptosis genes. In addition, CDDO-Im-mediated autophagy enhancement may also contribute to reduced apoptosis given previous studies that indicate the protection role of autophagy in blocking the onset of apoptosis. Necrosis cells dump tons of DAMPs to further stimulate liver inflammatory responses, which could lead to inflammation-induced secondary liver injury. In present study, we demonstrate that CDDO-Im-mediated autophagy enhancement accounts for its protection on necrosis. By elevating autophagy activity, CDDO-Im augments the clearance of damaged mitochondria, prevents the excessive ROS production and ATP depletion, resulting in reduced cell necrosis.

Autophagy is generally recognized as a cytoprotective mechanism against multiple cellular stresses through its functions including degradation of long-lived cytosolic and damaged proteins, clearing up of damaged mitochondria and modulation of cell death.^19, 29^ The protective effects of autophagy during the pathologic process of I/R has been identified by several studies.^22, 30, 31, 32, 33^ In present study, we found enhanced autophagy activity in CDDO-Im group as evidenced by increased expression of LC3B-II and decreased expression of SQSTM1. This enhanced autophagy contributes to CDDO-Im-related protection on liver I/R injury because the use of an autophagy inhibitor, 3-MA, almost completely abolished such protection.

A previous study demonstrates that Nrf2 can strengthen autophagy by directly upregulating the expression of Atg3, Atg6 and Atg12 in tumor cells.^34^ However, CDDO-Im-mediated autophagy enhancement is unseemly through the upregulation of those genes. HO-1 as a downstream target gene of Nrf2 has been implicated in protection of hepatic injury during infection/sepsis.^35^ By applying Nrf2 KO mice, we identified the essential role of Nrf2/HO-1 pathway in CDDO-Im-mediated autophagy. Furthermore, by using Nrf2 RNAi and SnPP, an inhibitor of HO-1, we demonstrate that the CDDO-Im-mediated hepatic protection and autophagy enhancement are dependent on Nrf2/HO-1 pathway. Although the mechanism by which HO-1 promotes autophagy activity is not characterized, we hypothesize that carbon monoxide, a product of HO-1 reaction, might be a signaling molecule to stimulate autophagy, because a recent report indicates that carbon monoxide can activate autophagy.^36^

Collectively, our present study indicates that CDDO-Im has a protective role on liver I/R injury by promoting Nrf2/HO-1-mediated autophagy activity, which results in increased clearance of damaged mitochondria, reduced production of ROS and inflammatory cytokines, and cell death. These findings strongly suggest a therapeutic value of CDDO-Im in liver I/R injury.

## Materials and methods

### Animals

Male WT C57BL/6 mice (8–10 weeks old) were purchased from Shanghai SLAC Co. Ltd (Shanghai, China). Nrf2 KO mice (8–10 weeks old, #017009 from Jackson Lab, Bar Harbor, ME, USA) were kindly provided by Dr Li-Wei Dong, an associate investigator, in the Second Military Medical University, Shanghai, China. All procedures involving animals were reviewed and approved by the Institutional Animal Care and Use Committee of the Shanghai Jiao Tong University School of Medicine (approval no. SYKX-2008-0050).

### Model of warm liver I/R injury

The model of partial hepatic I/R injury was used in our previous study.^22^ In brief, an atraumatic clip was used to interrupt the arterial and portal venous blood supply to the cephalad lobes of the liver for 90 min. Sham controls underwent the same procedure, but without vascular occlusion. CDDO-Im (2 mg/kg) (Tocris Bioscience, Bristol, UK) was administered intraperitoneally 3 h before onset of liver ischemia, whereas chloroquine (CQ, 60 mg/kg) or 3-Methyladenine (30 mg/kg) (Sigma-Aldrich, St. Louis, MO, USA) was administered intraperitoneally 1 h before the operation of liver ischemia. Tin Protoporphyrin IX dichlorid (SnPP) (50 mg/kg, Santa Cruz, Dallas, TX, USA) was administered intraperitoneally 1 h before injection of CDDO-Im or DMSO. In vehicle-treated mice, a volume of 0.5% DMSO or saline solution equal to that of treatment was administered in the same manner.

### Biochemical measurement

Blood was collected by direct puncture of arteriae aorta then centrifuged at 3000 × *g* for 5 min. Serum ALT and AST levels were measured by standard clinical automatic analyzer (Dimension Xpand; Siemens Dade Behring, Munich, Germany). MDA was measured by microplate test kits (Nanjing Jiancheng Bioengineering Institute, Nanjing, China) according to the manufacturer’s instructions.

### Liver histopathology, IHC analyses and TUNEL staining

Liver tissues were fixed in 4% paraformaldehyde for at least 24 h, then paraffin embedding through standard procedures and were cut into 5-*μ*m-thick sections. For liver histopathology, the sections were stained with hematoxylin and eosin. Suzuki’s criteria were used to evaluate the histological severity of liver injury.^37^ In brief, sinusoidal congestion, hepatocyte necrosis and ballooning degeneration were blindly graded from 0 to 4. For IHC analyses, liver sections were first rehydrated and processed for an antigen-unmasking procedure, and then incubated with primary antibodies against MPO (Cell Signaling, Boston, MA, USA), cleaved caspase-3 (Cell Signaling), F4/80 (AbD Serotec, Kidlington, UK) and hemeoxygenase-1 (HO-1) (Cell Signaling) overnight at 4 °C, followed by horseradish peroxidase-conjugated secondary antibodies. For histological analysis, sections were evaluated in a blinded manner by a pathologist. At least three fields per section were evaluated. TUNEL staining in liver sections was conducted by using an *In-Situ* Cell Death Detection Kit according to the manufacturer’s instructions (Roche Diagnostics, Indianapolis, IN, USA). For each stained section, at least three images from random fields were taken, and at least three mice per group were subjected to each experiment. Image-Pro Plus 6.0 was used for image analysis of sections.

### Hepatocyte isolation, culture and treatment

Primary hepatocyte isolation was performed as we previously described.^38^ The isolated cells were plated on dishes (3 × 10^6^ cells/6-cm dish), six-well plates (2 × 10^5^ cells/well) or 24-well plates (8 × 10^4^ cells/well). To simulate H/R *in vitro*, hepatocytes were cultured for 4 h at 37 °C in a modular incubator chamber (Biospherix, Lacona, NY, USA) gassed with 5% CO_2_, 90% N_2_ and 5% O_2_ for 4 h. For reoxygenation, hepatocytes were returned to the normoxic incubator for 2 h. To assess the cell injury, LDH was measured by LDH Release Assay Kit (Beyotime, Shanghai, China) according to the manufacturer’s protocols. Mitochondrial transmembrane potential in hepatocytes was assessed by ΔΨm assay kit with JC-1 (Beyotime) according to the manufacturer’s protocols. The decrease of ΔΨm was assessed by transition from JC-1 aggregates (Red) to JC-1 monomer (Green) in cells which indicating mitochondrial dysfunction and early cell death. Mitochondrial generation of superoxide was stained with MitoSOX Red (Invitrogen, Waltham, MA, USA). The Cyto-ID Autophagy Detection Kit (Enzo Life Sciences, Farmington, NY, USA) was used to identify the autophagosomes of the hepatocytes. In brief, after H/R injury, the hepatocytes were incubated in Cyto-ID Green stain solution in the dark for 30 min at 37 °C. Then analyze the stained cells by fluorescence microscope.

### Quantitative RT-PCR

Total liver RNA was extracted using TRIzol (Takara, Tokyo, Japan) reagent according to the manufacturer’s instructions. The cDNA was synthesized using 1000 ng of total RNA in the first-strand cDNA synthesis reaction with PrimeScript RT reagent Kit (Takara). RT-PCR was performed using the CFX 96 q-PCR system (BIO-RAD, Hercules, CA, USA). A SYBR RT-PCR kit (Takara) were used for quantitative real-time RT-PCR analysis. All reactions were conducted in a 20 *μ*l reaction volume in triplicate. The relative expression levels for a target gene were normalized by *β*-actin. Primers used for RT-PCR analysis are:

*Gclc* forward: 5′- ATGTGGACACCCGATGCAGTATT

*Gclc* reverse: 5′-TGTCTTGCTTGTAGTCAGGATGGTTT

*Gclm* forward: 5′- TGGAGCAGCTGTATCAGTGG

*Gclm* reverse: 5′- AGAGCAGTTCTTTCGGGTCA

*Gstm1* forward: 5′- CTACCTTGCCCGAAAGCAC

*Gstm1* reverse: 5′- ATGTCTGCACGGATCCTCT

*Nqo1* forward: 5′- AGCGTTCGGTATTACGATCC

*Nqo1* reverse: 5′- AGTACAATCAGGGCTCTTCTCG

*Il1b* forward: 5′- TGTAATGAAAGACGGCACACC

*Il1b* reverse: 5′- TCTTCTTTGGGTATTGCTTGG

*Tnf* forward: 5′- TTCTATGGCCCAGACCCTCA

*Tnf* reverse: 5′- TTTGCTACGACGTGGGCTAC

*Il6* forward: 5′- GCTACCAAACTGGATATAATCAGGA

*Il6* reverse: 5′- CCAGGTAGCTATGGTACTCCAGAA

*Cxcl-10* forward: 5′- GCTGCCGTCATTTTCTGC

*Cxcl-10* reverse: 5′- TCTCACTGGCCCGTCATC

*Actb* forward: 5′- TGACAGGATGCAGAAGGAGA

*Actb* reverse: 5′- ACCGATCCACACAGAGTACT.

### Western blots

Liver tissues were processed to western blot analysis as we previously described.^38^ Nuclear extracts were isolated from the livers or cultured hepatocytes, using NE-PER Nuclear and Cytoplasmic Extraction Reagents (Thermo Fisher scientific, Waltham, MA, USA). The primary antibodies against Bcl2, Bcl-XL, cleaved caspase-3, HO-1, LC3B, SQSTM1 (Cell Signaling), Nrf2 (Abcam), *β*-actin (Sigma-Aldrich) were used.

### TEM analysis

The liver tissues were fixed with 2.5% glutaraldehyde Ultrathin sections were cut and doubly stained with uranyl acetate and lead citrate. For autophagic vacuole quantification, 10 micrographs, primary magnification × 15 000, were randomly taken from each sample and the total amount of autophagic vacuoles was counted.

### Enzyme-linked immunosorbent assay (ELISA)

ELISA kits were used to detect mouse serum IL-6, TNF*α* (NeoBioscience Technology, Shenzhen, China) according to the manufacturer’s protocols.

### Caspase-3 activities

Caspase-3 activities were detected in liver tissues. The activities were measured with caspase-3 assay kit (Jiancheng Biotechnology, Nanjing, China) according to the manufacturer’s instructions.

### RNA interference in primary hepatocytes

Double-stranded siRNA corresponding to homologous sequence of mouse Nrf2 gene (Santa Cruz Biotechnology, Dallas, TX, USA) and HO-1 gene (Gene Pharma, Shanghai, China) were used to inhibit Nrf2 and HO-1 expression. Transfection was conducted by using Lipofectamine RNAiMAX reagent (Life Technologies, Co., Grand Island, NY, USA) following the manufacturer’s instructions. A scrambled siRNA was transfected as the negative control. After transfection for 48 h, primary hepatocytes were followed by different drugs treatment and H/R injury for further study.

### mtDNA detection

mtDNA was measured by absolute quantitative real-time PCR as describe.^39^ Total DNA was isolated from plasma samples using QIAamp Blood and Mini Kit (Qiagen, Valencia, CA, USA), which removes plasma components with the potential to interfere in PCR analysis. The samples were then diluted, and the same amount of total DNA was added to each reaction on each plate. The amount of mtDNA was determined using a pair of primers targeting mouse cytochrome c oxidase subunit III (Forward: 5′-ACCAAGGCCACCACACTCCT-3′ and Reverse: 5′-ACGCTCAGAAGAATCCTGCAAAGAA-3′). To construct standard curves, mitochondrial pellets were isolated from mouse liver by differential centrifugation. Purity of mtDNA standards was verified by real-time PCR using primers for both mitochondrial genes and nuclear-encoded *β*-actin. Dilutions of these purified mtDNA samples were prepared and standards were included on each PCR plate for each gene tested. The limit of detection for the assay was determined to be <0.05 ng/ml.

### Statistical analysis

Data were expressed as mean±S.E.M. Statistical significance was determined by two tailed, unpaired or paired Student’s *t*-test. A *P*-value<0.05 was used to indicate a statistically significant difference in all statistical comparisons,
