# Supplementary figures and images for: The triterpenoid CDDO-imidazolide ameliorates mouse liver ischemia-reperfusion injury through activating the Nrf2/HO-1 pathway enhanced autophagy
Source: Cell Death Dis. 2017 Aug 10;8(8):e2983–. doi: 10.1038/cddis.2017.386 (PMC5596572; doi:10.1038/cddis.2017.386)

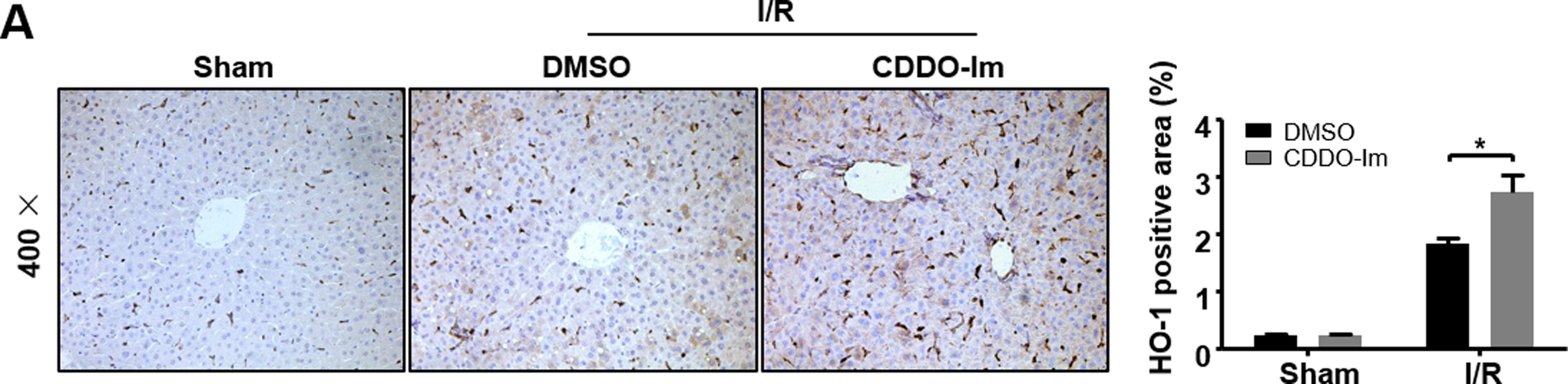

Supplement: Supplementary Figure S1 [file cddis2017386x1.tif]

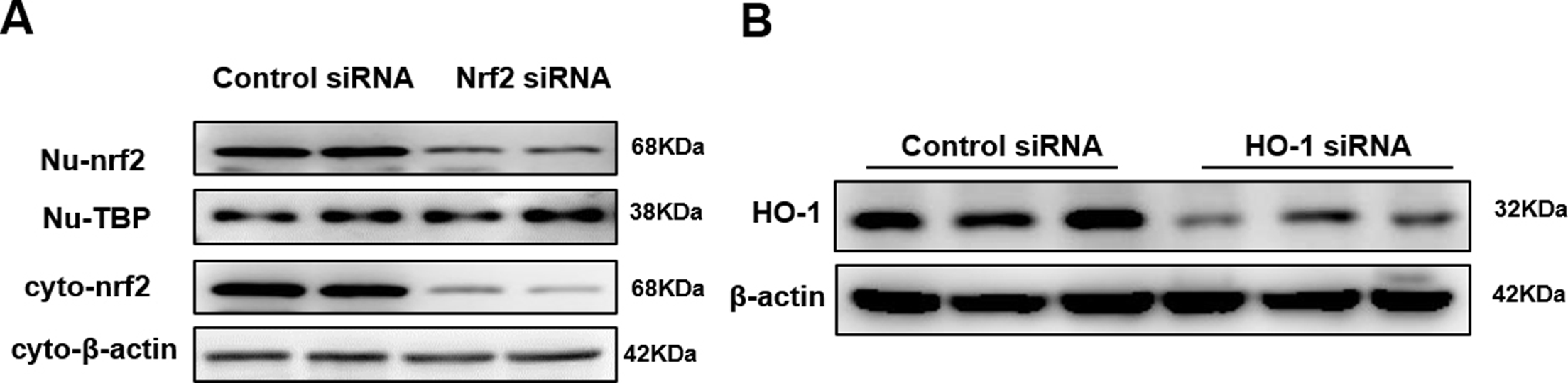

Supplement: Supplementary Figure S2 [file cddis2017386x2.tif]
