# Supplementary material for: The triterpenoid CDDO-imidazolide ameliorates mouse liver ischemia-reperfusion injury through activating the Nrf2/HO-1 pathway enhanced autophagy
Source: Cell Death Dis. 2017 Aug 10;8(8):e2983–. doi: 10.1038/cddis.2017.386 (PMC5596572; doi:10.1038/cddis.2017.386)
Supplement: Supplementary Figure Legends [file cddis2017386x3.docx]

**Supplementary Figure Legends**

**Figure S1.** (A) Representative sections from HO-1 staining (original magnification, 400×), the HO-1 positive area that infiltrated the livers were determined. (n=3-4 per group, mean ± SEM, **P<0.01, *P<0.05).

**Figure S2.** (A) Western blot indicating expression of Nrf2 protein levels in both nucleus and cytoplasm after treatment with Nrf2 siRNA for 48 h. (B) Western blot indicating expression of HO-1 protein after treatment with HO-1 siRNA for 48 h.
